# A newly described imaging finding for idiopathic normal pressure hydrocephalus: Can hummingbird sign contribute to the diagnosis?

**DOI:** 10.3906/sag-2107-86

**Published:** 2021-09-25

**Authors:** Başak ATALAY, Umut Perçem ORHAN SÖYLEMEZ, Hüseyin YILDIZ

**Affiliations:** 1 Department of Radiology, Faculty of Medicine, İstanbul Medeniyet University, İstanbul, Turkey; 2 Department of Radiology, Faculty of Medicine, İstanbul Medeniyet University, Göztepe Süleyman Yalçın City Hospital, İstanbul, Turkey

**Keywords:** Idiopathic normal pressure hydrocephalus, hummingbird sign, progressive supranuclear palsy, magnetic resonance imaging, mesencephalon

## Abstract

**Background/aim:**

In this study, we aimed to evaluate whether morphological changes in the mesencephalon, which were previously described as a diagnostic tool for progressive supranuclear palsy, could be associated also with idiopathic normal pressure hydrocephalus.

**Materials and methods:**

Consecutive 52 patients with a possible diagnosis of idiopathic normal pressure hydrocephalus (32 female, mean age 73.6 years) and 40 controls (23 female, mean age 72.7 years) with similar demographic characteristics were included the study. The morphologic changes in mesencephalon, hummingbird sign, and the vascular compression to mesencephalon were noted. Besides, three independent observers evaluated the imaging parameters for idiopathic normal pressure hydrocephalus in magnetic resonance imaging. Inter-observer reliabilities for qualitative and quantitative data were assessed using the Cronbach’s alpha and intra class correlation coefficient. The correlation of the imaging parameters with each other was evaluated with Pearson correlation.

**Results:**

Hummingbird sign was found to be significantly more common among patients with idiopathic normal pressure hydrocephalus (p < 0.0001). A statistically significant correlation was found between hummingbird sign and vascular compression of patients in the study group (p < 0.0001). A substantial, good, and perfect agreement was found between observers at all levels except callosal angle (fair agreement).

**Conclusion:**

Hummingbird sign can be used to support the diagnosis of idiopathic normal pressure hydrocephalus in addition to other radiological findings. A significant correlation between vascular compression and hummingbird sign in the patient group may explain the morphological changes in the mesencephalon that resemble the Hummingbird sign, which was previously described for progressive supranuclear palsy.

## 1. Introduction

Idiopathic normal pressure hydrocephalus (iNPH) is the most common form of hydrocephalus in adults. Its prevalence is 0.2% between the ages of 70–79 [1]. This is a condition accompanied with cognitive dysfunction, gait disturbance, and urinary incontinence. iNPH is a progressive neurological syndrome that etiology has not yet known. But the cerebrospinal fluid dynamics are disturbed, and ventricular dilatation occurs [2–6].The diagnosis of iNPH is based on typical clinical findings and magnetic resonance imaging (MRI), including dilatation of the temporal horns, acute callosal angle, increased T2 weighted (W) signal intensities in periventricular white matter, focally asymmetric enlarged sulci, disproportionate changes in subarachnoid spaces with dilated Sylvian fissures, and obliterated sulci and subarachnoid spaces at the convexity defined as disproportionately enlarged subarachnoid-space hydrocephalus (DESH) [7,8].

The only effective treatment method is known to be shunt surgery. However, it has been reported to be ineffective in some individuals [9]. Although many studies are reporting that imaging contributes to the diagnosis of iNPH can be used in the selection of appropriate candidates for shunt surgery, their prognostic role is still controversial. Agerskov et al. reported that imaging markers that may be valuable in the diagnosis of iNPH such as DESH sign and the acute callosal angle should not be used to exclude patients from shunt surgery. Besides, the authors remarked that these markers have no prognostic role in the reversibility of the symptoms [6].

iNPH can be confused particularly with atypical Parkinsonism because of the similar clinical spectrum. Symptoms such as gait dysfunction and postural retropulsion in progressive supranuclear palsy (PSP) may be confused with iNPH patients, with cognitive impairments that may be accompanying the symptoms. PSP is a progressive neurological disorder radiologically characterized by the presence of midbrain atrophy. Despite cardinal findings, vertical gaze palsy and ocular dysfunction may not be present in early PSP patients [10,11]. Similar morphological changes of mesencephalon including flattening, and concave appearance in the upper contour can be seen in midsagittal MR images in iNPH patients. From this point of view, we hypothesized that vascular compression and enlargement of third ventricle in iNPH patients may cause morphologic changes in mesencephalon resembling hummingbird sign.

## 2.Materials and methods

This study was approved by the University Ethics Committee for Medical Research (ID; 2020/0702), and, since it is retrospective, the informed consent was waived by the committee. Brain MRI performed between January 2019 and January 2021 was evaluated retrospectively.

A database search of medical records was conducted to select possible iNPH patients. After a primary search, duplicated patients were excluded. Patients with a possible diagnosis of iNPH were included in the study [12]. The diagnosis of iNPH was verified with imaging findings. Patients having inadequate images, with large infarct or tumor, without typical radiological findings of iNPH were excluded from the study. A total of 60 patients were evaluated. After exclusion, 52 patients were included in the study. A case match was performed, and an iNPH-free control group including 40 patients having similar demographic features was constituted.

### 2.1. Imaging protocols for MRI

MRI was performed using Optima 450w 1.5 T (GE Healthcare, Milwaukee, Wisc., USA) and included the following sequences: axial T1W (TR/TE, 540/12 ms) and T2W (TR/TE, 4640/89 ms) fast spin-echo, axial and coronal fluid-attenuated inversion-recovery (FLAIR) (TR/TE, 7000/92 ms), sagittal T2W (4025/106) fast spin-echo with a 5 mm slice thickness, DWI (TR/TE, 5900/98 ms; field of view, 250 × 250 mm; slice thickness: 5 mm; matrix, 128 × 128; b value: 0 and 1000 s/mm^2^).

### 2.2. Imaging parameters

Three observers with different experiences (seven and five years experienced specialist in neuroradiology and a senior resident) independently reviewed the radiological findings, blinded to the control and patient groups. Also, the association between MRI findings and evident clinical signs was evaluated.

**Figure 1:**
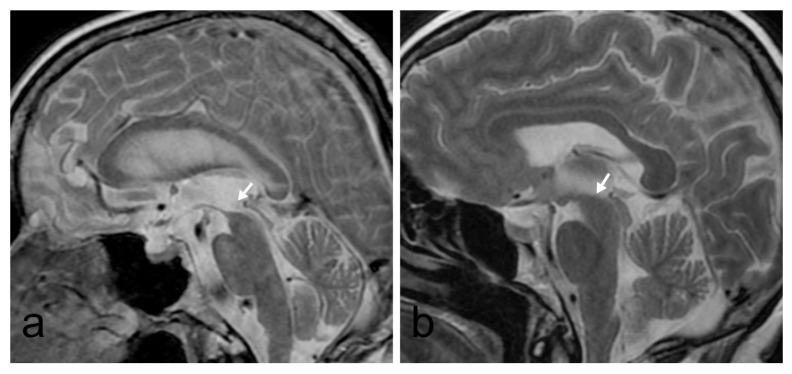
Sagittal T2W images. **1a**. In an iNPH patient, the concave appearance of the mesencephalon and vascular compression is shown. **1b**.The normal convex upper contour of mesencephalon in a control group patient.

Evans index (EI) is the ratio of the widest diameter of the frontal horns of the lateral ventricles to the maximum internal diameter of the skull at the cross-sectional level passing through the foramen Monroe. EI was measured on axial MRI. The third ventricle width was measured in millimeters as the widest diameter on axial MRI. The fourth ventricle width was measured on sagittal images as the widest anteroposterior midline diameter. The temporal horn width of lateral ventricles was measured on coronal images separately, and the mean value of two sides was noted. The interpeduncular angle (IPA) was measured at the level of the mamillary bodies on axial images. The callosal angle (CA) was measured on coronal images between the lateral ventricles through the posterior commissure, perpendicular to the anterior/posterior commissure line. The presence of one or more asymmetric sulcal widening on axial and coronal images was accepted as transport sulci. The presence of dilatation of Sylvian fissure was considered when they were wider than adjacent sulci. The presence of obliterated convexity sulcus with Sylvian fissure dilatation was noted as a DESH sign. The presence of flow void sign at cerebral aqueduct level on sagittal T2W images was noted. Periventricular white matter changes were evaluated on the FLAIR sequence and classified according to the Fazekas scale [13]. The presence of a tortuous posterior circulation was noted as vascular compression when the basilar or posterior cerebral artery touches the mamillary body. The flattening or concave counter of mesencephalon on midsagittal images was also noted as hummingbird sign (Figures 1a and 1b).

### 2.3. Statistics

Data were analyzed using SPSS software (ver. 22.0; IBM Corp., Armonk, NY, USA). Inter-observer reliabilities for qualitative data were assessed using Cronbach’s alpha (A). A value of 0.81 to 1.00 indicates almost perfect agreement, 0.61 to 0.80 indicates substantial agreement, 0.21 to 0.60 shows moderate agreement, and 0.20 or lower represents slight agreement. Intra class correlation coefficient (ICC) was used for the assessment of the reliability of the quantitative data between two observers. An ICC value of <0.50 indicates poor agreement, 0.50–0.75 indicates fair agreement, 0.75–0.90 indicates good agreement and 0.90–1 indicates excellent agreement [14]. The Mann–Whitney U test and Student t-test were used to compare quantitative data between two independent groups. Comparisons of categorical variables were performed using the Pearson chi-squared test. P-values <0.05 were considered to indicate statistical significance.

## 3. Results

Demographic data for the iNPH and control groups are reported in Table 1. Three major clinical symptoms were evident in only 9 (17%) patients.

A good agreement between observers was met considering EVANS index, third and fourth ventricles and temporal horn widths, and interpeduncular angle (ICC;0.787–0.889). A fair agreement in the measurement of callosal angle was met (ICC;0.525). A perfect agreement for the evaluation of Fazekas classification, transport sulci, obliterated sulci at the vertex, dilated Sylvian fissure, presence of DESH, flow void, and hummingbird sign (A;0.909-1), and a substantial agreement for the presence of vascular compression sign was met (A;0.751).

### 3.1. Imaging findings

**Figure 2a:**
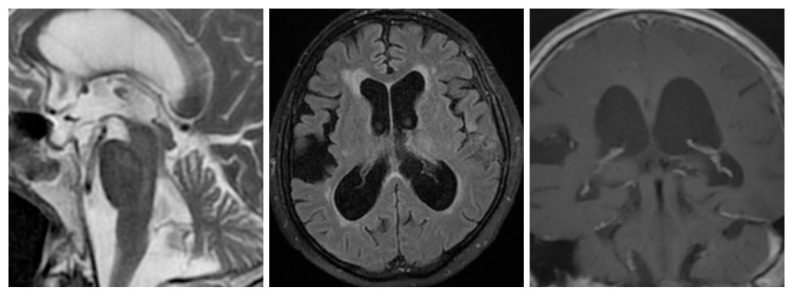
Sagittal T2W image; an iNPH patient the concave appearance of the mesencephalon and vascular compression is shown. **2b**. Axial FLAIR image; lateral ventricular dilatation and right-sided transport sulci and periventricular hyperintensities are present. **2c**. The coronal image shows the narrow callosal angle and dilated lateral ventricles.

A significant difference between two groups was detected for EVANS index, obliterated sulci at the vertex, presence of DESH sign, width of the third and fourth ventricle, temporal horn, interpeduncular angle, and callosal angle. The presence of hummingbird sign and presence of vascular compression were significantly more common in iNPH patients (Figure 2a, 2b and 2c). There was a high correlation with the presence of obliterated sulci and DESH sign (rho;0.700; p < 0.0001). The results of imaging markers are summarised in Table 2.

**Figure 3:**
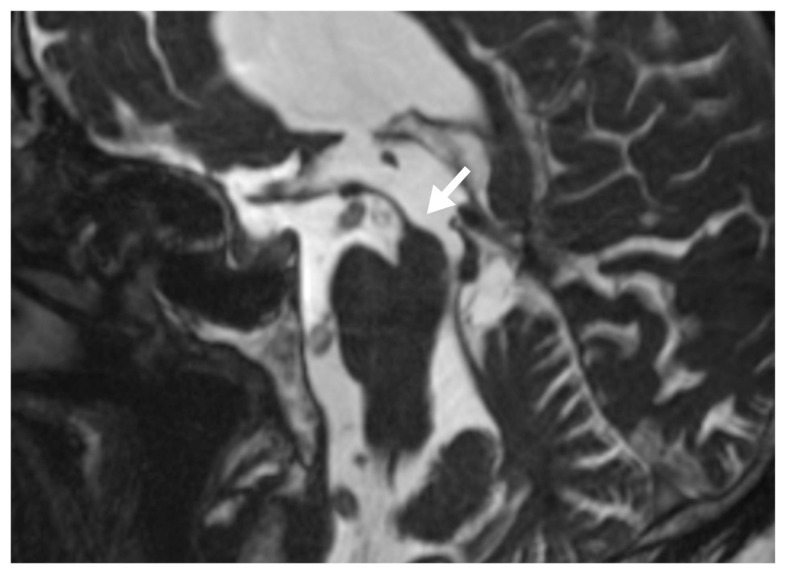
Sagittal T2W image, the convex appearance of the upper contour of the mesencephalon disappeared (arrow) in an iNPH patient and also the compression of the basilar artery to the floor of the third ventricle.

A statistically significant correlation was found between presence of hummingbird sign and vascular compression of the patients in the study group (rho;0.527; p <0.001). There was no statistically significant correlation between the presence of hummingbird sign and vascular compression findings in the control group (Figure 3). There was no significant correlation between the presence of hummingbird sign and third ventricle enlargement in both the study and control groups (Table 3).

The CA was found to be significantly narrower, and the third ventricle diameter was larger in patients who have three typical clinical symtoms (p = 0.042 and p = 0.040, respectively). However, there was no significant correlation between other parameters and manifestation of syptoms.

## 4. Discussion

In this study, we evaluated the MRI findings of iNPH and the morphological changes in the mesencephalon. The typical clinical findings of iNPH can be seen in the elderly population and PSP patients as well. Moreover, radiological findings can also be overlapped [10,11]. In the current study, a strong correlation between iNPH and EI, the width of third and fourth ventricles, and temporal horns, IPA, callosal angle, presence of DESH, and obliterated sulci at convexity was detected. Patients with typical clinical signs for iNPH had a narrower callosal angle and wider third ventricles.

We had hypothesized that vascular compression to mesencephalon and third ventricular dilatation may cause morphologic changes in the mesencephalon and resemble hummingbird sign. The comparison of the presence of hummingbird sign and vascular compression in the iNPH and control group showed that there are significant differences. In the patient group, there was a correlation between the hummingbird sign and vascular compression. Whereas, no correlation was found in the control group for these variables. Our results revealed that adding the evaluation of hummingbird sign to classical imaging findings may be also useful in the diagnosis of iNPH. We mentioned the morphological parameters in the diagnosis of iNPH and compared them with the control group with interobserver findings according to raise the reliability. Experience is essential for precise and accurate callosal angle measurements. In the current study, the lowest agreement was in the callosal angle (fair agreement). Hence, adding the evaluation of mesencephalon morphology may contribute to the diagnosis of iNPH.

While MRI is widely used to differentiate Parkinson’s disease and atypical Parkinson’s disease, there may be cases where the distinction of these neurodegenerative disorders is still insufficient due to overlaps in imaging findings [15–17]. Ugga et al. had evaluated the differences between iNPH, PSP, and healthy controls in terms of MRI findings [11]. The authors had found larger IPA values useful to help differentiation of iNPH from PSP and healthy controls. They mentioned that IPA values can be used in the diagnosis of iNPH like the narrow callosal angle. Similarly, we found wider IPA values in iNPH patients. The presence of a tortuous posterior circulation in iNPH patients has been claimed to cause a displacement to the floor of the third ventricle and might have produced a concave appearance in the upper mesencephalon [11]. In the evaluation of the diagnostic value and possible causes of hummingbird sign, we found that the changes in the mesencephalon were associated with vascular compression.

In a study conducted with a relatively small sample size of PSP, iNPH, and Alzheimer’s disease (AD) patients, the upper midbrain morphological changes and a tight appearance of the posterior part of the cingulate sulcus have been evaluated. While these signs wasnot observed in the control group, the cingulate sulcus sign was highly sensitive for iNPH. The upper midbrain profile sign had a sensitivity of 70% for iNPH and 60% for PSP. In this study, the hummingbird sign was defined in 7 out of 10 iNPH patients [18]. In a recent study, authors had reported hummingbird sign in 6 (35%) of 17 iNPH patients and 1 (3.4%) of 29 controls [19]. However, we found the hummingbird sign in 47 (90%) of 52 patients with a higher rate. In the control group, hummingbird sign was detected in 3 (7.5%) of 40 individuals. It could be speculated that the observers may behave biassed, but they were blind to control and patient group, and also statistical analyses revealed a perfect agreement between the 3 observers. The diagnosis and clinical significance of the presence of this finding should be investigated with studies to be conducted with larger series including different neurodegenerative diseases.

In a different case series authors had reported that midbrain diameter increased after ventriculoperitoneal shunting in 12 iNPH patients [20]. Whereas a study with 21 iNPH patients, after ventriculoperitoneal shunting, authors had reported no alteration in midbrain morphology [21]. Previously, a patient with gait disturbance had ventricular dilatation and a Hummingbird sign had been reported to be treated with shunt surgery. After the shunt surgery not only remediate the ventricular dilatation but also had vanished the Hummingbird sign. This can be explained by the disappearance of compression of the upper contour of the midbrain by the dilatation of third and lateral ventricles [22]. Similarly to this case report, we had hypothesized that these morphological changes in the upper mid-brain may be due to the compression of the expansion in the third ventricle. However, probably due to the limited number of cases included, our results revealed no evidence to support our hypothesis. This finding may have been due to displacement of the floor of the third ventricle upwards by vascular compression.

The major limitation of the study was the retrospective design and relatively small number of cases. The relatively higher number of female patients in the study group was another limitation. Future multicentric randomized controlled trials with larger patient groups with extrapyramidal diseases such as PSP, in which morphological changes in the mesencephalon are observed, are needed to drive exact conclusions. Another limitation was that it was not known whether the patients we included in the study have undergone shunt surgery or not. Hence, the prognostic role of newly described Hummingbird sign in treatment is unpredictable due to unknown treatment results of iNPH patients.

## 5.Conclusion

The tortuous course of the posterior circulation and vascular compression may cause upper displacement of the floor of the third ventricle and mimic hummingbird sign in iNPH patients. However, this is not true midbrain atrophy-hummingbird sign. DESH sign, narrow callosal angle, wide temporal horns are valuable for the diagnosis of iNPH, and these typical imaging findings may be accompanied by morphological changes in the mesencephalon. There is not any well-described imaging marker for the clinical outcome after external drainage. The newly described hummingbird sign for iNPH patients may be a supporting prognostic factor that future studies with prospective design and larger patient series would provide.

## Figures and Tables

**Table 1:** Age and sex distribution in the study and control groups.

	Patient group (n = 52)	Control group (n = 40)	p value
Sex (F/M)	(32/20)	(23/17)	0.151^Q^
Age(mean/±SD/median/IQR)	73.6/±7.96/73/9	72.7/±6.26/71/8	0.65 U

U ; Mann–Whitney u test,

q ; Chi-Square test, IQR: interquartile rate.

**Table 2:** Comparison of the evaluated parameters in the patient and control groups.

		Patient group (n = 52)	Control group (n = 40)	p value
Fazekas	0	1 (1.9%)	9 (22.5%)	**0.231** ^P^
	1	10 (19.2%)	22 (55%)	
	2	28 (53.8%)	7 (17.5%)	
	3	13 (25%)	3 (3.5%)	
Periventricular hyperintensity		48 (92.3%)	12 (30%)	**0.527** ^P^
Transported sulci		38 (73.1%)	5 (2.5%)	**0.503** ^P^
Obliterated sulci at vertex		50 (96.2%)	0 (0%)	**<0.00001** ^P^
Dilated slyvian fissure		48 (92.3%)	20 (50%)	**0.292** ^P^
DESH present		51 (98.1%)	0 (0%)	**<0.00001** ^P^
Flow void sign		22 (42.3%)	3 (7.5%)	**0.278** ^P^
Humming bird		48 (92.3%)	3 (7.5%)	**<0.00001** q
Vascular compression		40 (76.9%)	18 (45%)	**0.925** ^P^
EVANS index(Mean/range/±SD)		0.35 (0.29–0.44) ± 0.03	0.24 (0.2–0.3) ± 0.02	**0.0135** ^u^
Third ventricle width (mm)(Mean/range/±SD)		13.1 (8.2–22.8) ± 3	6.2 (3.5–9.2) ± 1.3	**<0.00001** ^u^
Temporal horn width (mm)(Mean/range/±SD)		6.5 (2–14.2) ± 2.3	1.9 (1.1–4.4) ± 0.7	**<0.00001** ^u^
Fourth ventricle width(mm)(Mean/range/±SD)		11.7 (8–15.9) ± 1.8	9.9 (7.7–12) ± 7.8	**0.0394** ^u^
Interpedincular angle (degree)(Mean/range/±SD)		90.1 (71.1–107) ± 7.1	82.8 (67–99) ± 7.9	**0.0394** ^u^
Callosal angle (degree)(Mean/range/±SD)		77.3 (47.6–99) ± 11.6	122.7 (108–139.9) ± 7.8	**<0.00001** ^u^

U ; Mann Whitney U test,

q ; Chi-Square test,

DESH: disproportionally enlarged subarachnoid space hydrocephalus.

**Table 3:** Correlation of the evaluated parameters with each other.

	Patients with three clinical symptoms	Vascular compression	Humming bird sign	Flow void	DESH	Dilated sylvian fissure	Obliterated sulci	Transported sulci	Periventricular hyperintensity	Fazekas

**Patients with three clinical symptoms (rho,p)**		0.130	0.132	0.123	0.064	0.132	0.091	0.066	0.132	0.199
	1	0.359	0.351	0.386	0.652	0.351	0.519	0.641	0.351	0.156

**Vascular compression (rho,p)**	0.130	1	**0.527** **	0.099	0.256	0.013	0.128	0.079	0.013	0.331*
	0.359		**0.000**	0.483	0.067	0.926	0.367	0.577	0.926	0.016

**Humming bird sign (rho,p)**	0.132	**0.527** **	**1**	0.247	0.040	0.188	0.058	0.150	0.188	0.208
	0.351	**0.000**		0.077	0.776	0.183	0.684	0.288	0.183	0.139

**Flow void (rho,p)**	0.123	0.099	0.247	1	0.164	0.247	0.031	0.095	0.045	0.031
	0.386	0.483	0.077		0.247	0.077	0.827	0.505	0.752	0.826

**DESH (rho,p)**	0.064	0.256	0.040	0.164	1	0.040	**0.700** **	0.231	0.040	0.004
	0.652	0.067	0.776	0.247		0.776	**0.000**	0.100	0.776	0.979

**Dilated sylvian fissure (rho,p)**	0.132	0.013	0.188	0.247	0.040	1	0.318*	0.313*	0.188	0.008
	0.351	0.926	0.183	0.077	0.776		0.022	0.024	0.183	0.957

**Obliterated sulci (rho,p)**	0.091	0.128	0.058	0.031	**0.700** **	0.318*	1	0.330*	0.058	0.005
	0.519	0.367	0.684	0.827	**0.000**	0.022		0.017	0.684	0.970

**Transported sulci (rho,p)**	0.066	0.079	0.150	0.095	0.231	0.313*	0.330*	1	0.013	0.016
	0.641	0.577	0.288	0.505	0.100	0.024	0.017		0.930	0.909

**Periventricular hyperintensity (rho,p)**	0.132	0.013	0.188	0.045	0.040	0.188	0.058	0.013	1	0.308*
	0.351	0.926	0.183	0.752	0.776	0.183	0.684	0.930		0.026

**FAZEKAS (rho,p)**	0.199	0.331*	0.208	0.031	0.004	0.008	0.005	0.016	0.308*	1
	0.156	0.016	0.139	0.826	0.979	0.957	0.970	0.909	0.026	

rho: Pearson correlation coefficiant.

p;

** Correlation is significant at the 0.01 level

* Correlation is significant at the 0.05 level.
